# The transmissibility estimation of influenza with early stage data of small-scale outbreaks in Changsha, China, 2005–2013

**DOI:** 10.1017/S0950268816002508

**Published:** 2016-11-11

**Authors:** T. M. CHEN, Q. P. CHEN, R. C. LIU, A. SZOT, S. L. CHEN, J. ZHAO, S. S. ZHOU

**Affiliations:** 1 Department of Malaria, National Institute of Parasitic Diseases, Chinese Center for Disease Control and Prevention, Shanghai, People's Republic of China; 2 Changsha Center for Disease Control and Prevention, Changsha, Hunan, People's Republic of China; 3 Hospital, Shanghai Normal University, Shanghai, People's Republic of China; 4 Mass Medical International, Brookline, MA, USA

**Keywords:** Influenza, mathematical model, reproduction number, small-scale outbreak

## Abstract

Hundreds of small-scale influenza outbreaks in schools are reported in mainland China every year, leading to a heavy disease burden which seriously impacts the operation of affected schools. Knowing the transmissibility of each outbreak in the early stage has become a major concern for public health policy-makers and primary healthcare providers. In this study, we collected all the small-scale outbreaks in Changsha (a large city in south central China with ~7·04 million population) from January 2005 to December 2013. Four simple and popularly used models were employed to calculate the reproduction number (*R*) of these outbreaks. Given that the duration of a generation interval *Tc* = 2·7 and the standard deviation (s.d.) *σ* = 1·1, the mean *R* estimated by an epidemic model, normal distribution and delta distribution were 2·51 (s.d. = 0·73), 4·11 (s.d. = 2·20) and 5·88 (s.d. = 5·00), respectively. When *Tc* = 2·9 and *σ* = 1·4, the mean *R* estimated by the three models were 2·62 (s.d. = 0·78), 4·72 (s.d. = 2·82) and 6·86 (s.d. = 6·34), respectively. The mean *R* estimated by gamma distribution was 4·32 (s.d. = 2·47). We found that the values of *R* in small-scale outbreaks in schools were higher than in large-scale outbreaks in a neighbourhood, city or province. Normal distribution, delta distribution, and gamma distribution models seem to more easily overestimate the *R* of influenza outbreaks compared to the epidemic model.

## INTRODUCTION

Each year, many countries or regions experience a peak influenza virus activity. A large number of people suffer from the infection and many individuals are affected by small-scale outbreaks in schools. There are hundreds of such small-scale influenza outbreaks in mainland China every year, leading to a heavy disease burden which seriously impacts the operation of affected schools. Knowing the transmissibility of each outbreak in early stages has become a major concern for the public health policy-makers and primary healthcare providers. Thus, it is necessary to estimate the reproduction number *R* (a most commonly used indicator that quantifies the transmissibility of influenza) when the primary health departments receive an outbreak report. However, the *R* of small-scale outbreaks has not yet been well estimated in China.

*R* is defined as the average number of secondary infections that arise from a typical primary case [1, 2]. From this definition, it is immediately clear that when *R* > 1, the disease is able to spread in the susceptible population. If *R* < 1, the infection will be cleared from the population. An individual-based model [3–5] or ordinary differential equation (ODE) model [2, 6] has been commonly employed for the estimation of *R*. In these models, the natural history of influenza (the incubation period, the latent period, infectious, or recovered, etc.), the demographic characteristics of the affected population, the epidemic data, and the counter-measures for controlling the outbreak are always included, which press the model closer to the actual scenarios hiding below the outbreak. Unfortunately, because of the professional gap between public health and the mathematical model, it is difficult for the primary health department in China to estimate the *R* of an outbreak by using an individual-based model or an ODE model. It is imperative to confirm a practical and accurate transmissibility estimation method for the primary health department.

There are several simple approaches which could be used for the estimation of *R* from epidemiological data. These are defined as ‘susceptibles at endemic equilibrium’, ‘average age at infection’, ‘the final size equation’, ‘calculation from the intrinsic growth rate’ by Heffernan *et al.* [7]. The epidemic model, normal distribution, delta distribution, and gamma distribution models by Wallinga *et al.* [8], Heffernan *et al.* [7] and Trichereau *et al.* [9] were also used. Heffernan's former three models are not easily used at the early stage of an outbreak because the parameters of these models are hard to obtain at that stage. The model ‘calculation from the intrinsic growth rate’ is the same as the epidemic model. Therefore, the epidemic model, along with the normal distribution, delta distribution, and gamma distribution models are the best choices for the model screening.

In this study, we collected all the small-scale outbreaks in Changsha from January 2005 to December 2013. The four simple and commonly used models [7–9] were employed to calculate the *R* values of these outbreaks, and the results of each model were compared in order to commend an optimized model which could be used. We also collected the data of interventions employed in each outbreak and calculated the reproductive number with control measures (*R*_con_) of each outbreak to discover the effectiveness of the interventions.

## METHODS

### Data collection

In China, the criterion for an influenza outbreak has been defined as ⩾10 influenza-like illness (ILI) cases occurring in the same school, preschool, or other collective organization within 1 week, with laboratory-confirmed influenza viruses through virus isolation or real-time reverse transcriptase–polymerase chain reaction analysis. Our study subjects also included public health incidents that were defined as ⩾30 ILIs within 1 week. ILI refers to a fever (axillary temperature ⩾38 °C) accompanied by coughing or sore throat and a lack of a laboratory-confirmed diagnosis of the specific pathogen.

We built a dataset of seasonal influenza and influenza A(H1N1)pdm outbreaks by collecting information on all outbreaks reported from 1 January 2005 to 31 December 2013 in Changsha, China. Data included location type (e.g. primary school, middle school, high school, and prison), the school population, the date of the reported outbreak, the date of symptom onset of all cases, the subtype of influenza virus, and interventions including symptom surveillance, case isolation, symptomatic treatment of cases, antivirals for treatment or prophylaxis use, health education, environment disinfection, vaccination, social distance, and class, grade and school closure.

Symptom surveillance which focused on ILI cases was implemented every day from the reported outbreak date to the end of the outbreak. For case isolation, infected individuals were isolated in the hospital if they reported severe symptoms like pneumonia, or were isolated at home for mild cases until all the symptoms disappeared after 24 h or 48 h. For symptomatic treatment, cases were treated by medication (not antivirals) to relieve the symptoms in the hospital or at home.

Local CDC staff had overseen health education for the affected people who were taught to maintain personal hygiene and wear a gauze mask during the outbreak. Local public health providers also disinfected the potential environment or air contaminated by cases, and a chlorine-based disinfectant was employed to sterilize the fomites and a peracetic acid solution of 15% concentration was employed to disinfect the air through fumigation.

During the period of the class/grade/school closure, a teacher in charge of a class was required to monitor all the students in the class every day. At the same time, each student was asked to stay at home, take their own temperature and report their findings to their teacher by telephone.

Two medications (moroxydine and a traditional medicine called ‘Ban Lan Gen Chong Ji’) were used for prophylaxis. Moroxydine was prescribed at 0·1 g t.i.d. for 2 or 3 days for children aged <10 years and the dose was doubled for those aged >10 years. ‘Ban Lan Gen Chong Ji’, which is made from an herbaceous plant named Ban Lan Gen, was used at 10 g t.i.d. for 5 days.

In our study, 56 influenza outbreaks in school, prison and the community were collected (Table 1). To make the estimation of *R* more stable and reliable, the inclusion criterion was that the exponential growth phase of outbreak should be ⩾3 days. The exclusion criteria were as follows: (*a*) that the outbreaks had no illness onset date of each case, or only a part of the cases had an onset date, which may make the epidemic curve of the outbreak unavailable for the estimation of *R*; (*b*) that there was no influenza laboratory test results in the outbreak; (*c*) there was a combined infection of influenza subtypes/types in an outbreak; and (*d*) the virus was untyped. According to these criteria only the target datasets would be included in our study for estimating the *R* of each outbreak.

**Table 1.** General information of 56 influenza outbreaks in Changsha city, China, 2005–2013 (n = 56)

Outbreak ID	Year	Month	Type of locations	Population	Accumulative cases	DO	Subtypes
1	2009	10	Secondary school	1101	44	14	H1N1pdm
2	2009	11	Secondary school	4644	143	11	H1N1pdm
3	2009	11	Secondary school	1811	77	12	H1N1pdm
4	2009	11	Primary school	1231	71	11	H1N1pdm
5	2009	11	Primary school	1028	58	21	H1N1pdm
6	2009	11	Secondary school	1874	38	10	H1N1pdm
7	2009	11	Secondary school	1192	59	15	H1N1pdm
8	2009	11	Secondary school	1342	256	40	H1N1pdm
9	2009	11	College	1357	61	23	H1N1pdm
10	2009	10	Training school	126	58	9	H1N1pdm
11	2009	10	Primary school	1129	107	14	H1N1pdm
12	2009	9	College	6546	114	22	H1N1pdm
13	2009	10	College	13 485	163	9	H1N1pdm
14	2009	11	Prison	694	83	17	H1N1pdm
15	2009	11	Primary school	264	66	11	H1N1pdm
16	2009	11	Secondary school	1240	100	11	H1N1pdm
17	2009	11	Secondary school	2050	101	14	H1N1pdm
18	2009	11	Secondary school	1138	93	15	H1N1pdm
19	2009	11	Primary school	1563	42	17	H1N1pdm
20	2009	11	Secondary school	4673	17	13	H1N1pdm
21	2009	11	Secondary school	1950	18	4	H1N1pdm
22	2009	10	Secondary school	4290	31	10	H1N1pdm
23	2009	10	Secondary school	2670	95	17	H1N1pdm
24	2009	9	Secondary school	4297	89	19	H1N1pdm
25	2009	10	College	2477	43	7	H1N1pdm
26	2009	9	College	1434	48	13	H1N1pdm
27	2009	11	Primary school	1081	155	21	H1N1pdm
28	2009	10	Secondary school	588	37	15	H1N1pdm
29	2009	11	Primary school	429	19	10	H1N1pdm
30	2009	11	Secondary school	2280	96	43	H1N1pdm
31	2009	11	Secondary school	1251	127	20	H1N1pdm
32	2009	11	Secondary school	1562	121	25	H1N1pdm
33	2009	11	Secondary school	5313	11	21	H1N1pdm
34	2009	11	Secondary school	2396	791	9	H1N1pdm
35	2010	1	Prison	628	86	12	H1N1pdm
36	2009	9	Secondary school	4032	60	15	H1N1pdm
37	2009	11	College	2255	49	15	H1N1pdm
38	2013	1	Secondary school	2500	80	19	H1N1pdm
39	2013	11	Secondary school	1490	44	15	H3N2
40	2013	11	Secondary school	4872	74	17	H3N2 + B
41	2009	8	Community	1900	41	10	H3N2
42	2009	2	Secondary school	7716	336	23	H1N1
43	2009	3	Primary school	671	43	15	B
44	2009	3	Primary school	885	43	13	H1N1 + B
45	2009	3	Secondary school	639	39	8	H1N1
46	2009	3	Secondary school	855	32	7	H1N1
47	2008	2	Primary school	160	28	9	H3N2
48	2008	6	Primary school	125	51	20	B
49	2007	3	Primary school	375	58	15	A (untyped)
50	2007	3	Secondary school	1539	42	9	A (untyped)
51	2006	4	Primary school	110	47	n.a.	Untyped
52	2006	2	Primary school	570	96	11	A (untyped)
53	2006	2	Primary school	187	58	10	A (untyped)
54	2006	2	Secondary school	210	47	13	A (untyped)
55	2005	12	Secondary school	1043	49	9	A (untyped)
56	2005	3	Primary school	317	27	n.a.	n.a.

ID, Identification; DO, duration of outbreak; n.a., not available.

### Estimation of reproduction number

To estimate the *R* of each outbreak, we employed the intrinsic growth rate method using the following four models [7–9].

#### Epidemic model

In this model, the reproduction number, *R*, is expressed as follows:

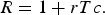


In this equation, *r* and *Tc* refer to the epidemic growth rate and the duration of a generation interval.

#### Normal distribution

In this model, the generation intervals may approximate a normal distribution, making *R* as follows:

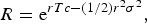

where *Tc* refers to the mean generation interval and *σ* refers to the standard deviation (s.d.).

#### Delta distribution

In this model, all secondary infections are exactly equal to the mean generation interval *Tc*. The model is expressed as follows:

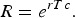


#### Gamma distribution

For the gamma distribution Gamma (*α, β*), the relationship between *r* and *R* is expressed as follows:

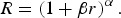

During the exponential growth phase of an outbreak, the relationship between the growth rate *r* and daily incidence *I*(*t*) of symptom onset can be expressed as d*I*/d*t* = *rI*, where *r* is the exponential growth rate [7, 10]. The exponential growth rate *r* was estimated from the daily epidemic curve of each outbreak we collected.

As *Tc* could not be estimated from our epidemic data, we used two different generation intervals derived from previous research [10, 11]. The mean generation intervals and standard deviations were *Tc* = 2·7, *σ* = 1·1 [11] and *Tc* = 2·9, *σ* = 1·4 [10]. The two parameters *α* and *β* of a gamma distribution Gamma (*α, β*) were 4·2 and 0·68, respectively [10]. The values of parameters (*Tc* = 2·9, *σ* = 1·4) and Gamma (4·2, 0·68) were calculated from the same data [10].

### Simulation methods

Berkeley Madonna v. 8.3.18 (University of California at Berkeley, USA) and Microsoft Office Excel 2003 (Microsoft, USA) software were employed for model simulation and figure development, respectively. The details of model-fitting methods run in Berkeley Madonna, such as the Runge–Kutta method of order 4 and root-mean-square deviation, were the same as the those in references [1, 2, 12].

### Ethical standards

The authors assert that all procedures contributing to this work comply with the ethical standards of the relevant national and institutional committees on human experimentation and with the Helsinki Declaration of 1975, as revised in 2008.

## RESULTS

After analysing 56 outbreaks, 32 outbreaks were enrolled for estimating *R*, because three outbreaks had no illness onset dates for each case and one had no laboratory test results. Moreover, the data of four outbreaks were not integrated, the combined infection of influenza subtypes/types were tested in two outbreaks, six outbreaks were untyped, and the exponential growth phase of nine outbreaks were <3 days. Of these 32 outbreaks, the growth rates of 15 were shown to have statistical significance by curve fitting (see Table 2). According to the generation interval used and its distribution, *R* estimations are shown in Table 3.

**Table 2.** The growth rate r of 32 influenza outbreaks in Changsha city, China, 2005–2013 (n = 32)

Outbreak ID	*r*			
	Value	95% CI	*R*^2^	*P* value
1	0·693	0·565–0·821	0·936	0·033
3	0·876	0·370–1·382	0·428	0·159
5	0·351	0·020–0·682	0·158	0·330
6	1·040	0·840–1·240	0·964	0·121
7	0·746	0·374–1·118	0·446	0·101
8	0·475	0·415–0·535	0·898	0·000
9	0·585	0·477–0·693	0·879	0·006
10	0·492	0·419–0·565	0·919	0·003
11	1·287	0·487–2·087	0·463	0·206
13	0·331	0·190–0·472	0·477	0·058
14	0·707	0·611–0·803	0·965	0·018
17	0·743	0·595–0·891	0·894	0·015
18	0·812	0·298–1·326	0·333	0·175
19	0·601	0·293–0·909	0·322	0·087
21	1·151	1·121–1·181	0·999	0·017
22	0·786	0·278–1·294	0·374	0·197
25	0·279	0·042–0·516	0·316	0·324
26	0·484	0·267–0·701	0·416	0·061
27	0·786	0·694–0·878	0·948	0·001
28	1·040	0·517–1·563	0·798	0·297
31	0·769	0·631–0·907	0·912	0·011
35	0·870	0·569–1·171	0·806	0·102
36	0·389	0·212–0·566	0·617	0·115
37	0·769	0·344–1·194	0·396	0·130
38	0·179	0·119–0·239	0·524	0·018
39	0·168	0·160–0·176	0·998	0·031
42	0·966	0·858–1·074	0·975	0·012
43	0·415	0·300–0·530	0·764	0·023
45	0·638	0·458–0·818	0·806	0·039
46	0·559	0·466–0·652	0·922	0·009
47	1·151	0·781–1·521	0·906	0·198
48	0·564	0·285–0·843	0·368	0·083

ID, Identification; *r*, epidemic growth rate; CI, confidence interval; *R*^2^, coefficient of determination.

**Table 3.** The reproduction number R of 15 influenza outbreaks in Changsha city, China, 2005–2013 (n = 15)

Outbreak ID	Epidemic model (*Tc* = 2·7)		Normal distribution (*Tc* = 2·7, *σ* = 1·1)		Delta distribution (*Tc* = 2·7)		Epidemic model (*Tc* = 2·9)		Normal distribution (*Tc* = 2·9, *σ* = 1·4)		Delta distribution (*Tc* = 2·9)		Gamma distribution Gamma(4·2,0·68)	
	*R*	95% CI	*R*	95% CI	*R*	95% CI	*R*	95% CI	*R*	95% CI	*R*	95% CI	*R*	95% CI
1	2·87	2·53–3·22	4·86	3·79–6·10	6·50	4·60–9·18	3·01	2·64–3·38	5·58	4·24–7·19	7·46	5·15–10·81	5·06	3·92–6·44
8	2·28	2·12–2·44	3·15	2·76–3·57	3·61	3·07–4·24	2·38	2·20–2·55	3·46	3·00–3·97	3·96	3·33–4·72	3·24	2·84–3·68
9	2·58	2·29–2·87	3·95	3·16–4·86	4·85	3·63–6·50	2·70	2·38–3·01	4·43	3·48–5·58	5·45	3·99–7·46	4·08	3·25–5·06
10	2·33	2·13–2·53	3·26	2·79–3·79	3·77	3·10–4·60	2·43	2·22–2·64	3·60	3·03–4·24	4·17	3·37–5·15	3·36	2·87–3·92
14	2·91	2·65–3·17	4·99	4·15–5·92	6·75	5·21–8·74	3·05	2·77–3·33	5·74	4·69–6·95	7·77	5·88–10·26	5·20	4·30–6·23
17	3·01	2·61–3·41	5·32	4·02–6·86	7·43	4·99–11·09	3·15	2·73–3·58	6·18	4·53–8·20	8·63	5·62–13·25	5·57	4·17–7·31
21	4·11	4·03–4·19	10·04	9·64–10·43	22·37	20·63–24·26	4·34	4·25–4·42	12·63	12·07–13·21	28·16	25·81–30·72	11·34	10·80–11·89
27	3·12	2·87–3·37	5·75	4·87–6·71	8·35	6·51–10·70	3·28	3·01–3·55	6·72	5·59–8·00	9·77	7·48–12·76	6·04	5·07–7·14
31	3·08	2·70–3·45	5·58	4·32–7·04	7·97	5·49–11·58	3·23	2·83–3·63	6·50	4·90–8·44	9·30	6·23–13·88	5·85	4·48–7·52
38	1·48	1·32–1·65	1·59	1·37–1·84	1·62	1·38–1·91	1·52	1·35–1·69	1·65	1·40–1·93	1·68	1·41–2·00	1·62	1·39–1·88
39	1·45	1·43–1·48	1·55	1·52–1·58	1·57	1·54–1·61	1·49	1·46–1·51	1·60	1·57–1·64	1·63	1·59–1·67	1·58	1·54–1·61
42	3·61	3·32–3·90	7·72	6·50–9·04	13·57	10·14–18·17	3·80	3·49–4·11	9·36	7·71–11·21	16·47	12·04–22·52	8·34	6·89–10·00
43	2·12	1·81–2·43	2·76	2·13–3·53	3·07	2·25–4·18	2·20	1·87–2·54	3·00	2·26–3·92	3·33	2·39–4·65	2·84	2·18–3·64
45	2·72	2·24–3·21	4·38	3·03–6·07	5·60	3·44–9·10	2·85	2·33–3·37	4·97	3·32–7·15	6·36	3·77–10·72	4·54	3·12–6·41
46	2·51	2·26–2·76	3·74	3·09–4·50	4·52	3·52–5·81	2·62	2·35–2·89	4·19	3·39–5·12	5·06	3·86–6·62	3·87	3·18–4·67

ID, Identification; *Tc*, mean generation interval; *σ*, standard deviation; *R*, reproduction number; CI, confidence interval.

Given that *Tc* = 2·7 and *σ* = 1·1, the mean *R* estimated by the epidemic model, normal distribution and delta distribution models were 2·68 (s.d. = 0·71), 4·58 (s.d. = 2·22) and 6·77 (s.d. = 5·28), respectively. Given that *Tc* = 2·9 and *σ* = 1·4, the mean *R* estimated by the three models were 2·80 (s.d. = 0·76), 5·31 (s.d. = 2·87) and 7·95 (s.d. = 6·73), respectively. The mean *R* estimated by gamma distribution was 4·84 (s.d. = 2·52) (see Table 3). The mean values of *R* estimated by normal distribution were higher than the those estimated by the epidemic model, even if *Tc* = 2·7 (*t* = −3·149, *P* = 0·006) or *Tc* = 2·9 (*t* = 3·265, *P* = 0·005). Similarly, the mean values of *R* estimated by delta distribution were higher than those estimated by the epidemic model, even if *Tc* = 2·7 (*t* = −2·974, *P* = 0·010) or *Tc* = 2·9 (*t* = −2·939, *P* = 0·011). However, there was no significance between the mean values of *R* estimated by delta distribution and those estimated by normal distribution, even if *Tc* = 2·7 (*t* = −1·483, *P* = 0·149) or *Tc* = 2·9 (*t* = −1·396, *P* = 0·174).

Because *Tc* = 2·9, *σ* = 1·4 and Gamma (4·2, 0·68) were from the same data, we only compared the results of the gamma distribution estimated with the results of the epidemic model, normal distribution and delta distribution models that had been tested under the same conditions. The mean *R* estimated by gamma distribution was higher than the mean *R* estimated by the epidemic model (*t* = −2·988, *P* = 0·008). However, the mean *R* estimated by gamma distribution was not significant compared to the mean *R* estimated by normal distribution (*t* = 0·478, *P* = 0·636) and delta distribution (*t* = 1·676, *P* = 0·105).

Of the 15 outbreaks, ten were H1N1pdm, one was H3N2, three were H1N1 and one was B subtype. There was no significance with the mean *R* of these subtypes based on the analysis of variance (ANOVA), even if *R* was estimated by the four models under different conditions (see Table 4).

**Table 4.** The results of ANOVA analysis in different subtypes of influenza virus (n = 15)

Models	Subtypes	No. of outbreaks	Mean of *R*	95% CI	*F* value	*P* value
Epidemic (*Tc* = 2·7)	H1N1pdm	10	2·78	2·29–3·27		
	H3N2	1	1·45	−		
	H1N1	3	2·95	1·50–4·40	1·591	0·247
	B	1	2·12	−		
	Total	15	2·68	2·29–3·07		
Normal distribution (*Tc* = 2·7, *σ* = 1·1)	H1N1pdm	10	4·85	3·24–6·46		
	H3N2	1	1·55	−		
	H1N1	3	5·28	0–10·59	0·988	0·434
	B	1	2·76	−		
	Total	15	4·58	3·34–5·81		
Delta distribution (*Tc* = 2·7)	H1N1pdm	10	7·32	3·23–11·41		
	H3N2	1	1·57	−		
	H1N1	3	7·90	0–20·18	0·509	0·684
	B	1	3·07	−		
	Total	15	6·77	3·85–9·69		
Epidemic (*Tc* = 2·9)	H1N1pdm	10	2·91	2·38–3·43		
	H3N2	1	1·49	−		
	H1N1	3	3·09	1·54–4·64	1·590	0·248
	B	1	2·20	−		
	Total	15	2·80	2·38–3·22		
Normal distribution (*Tc* = 2·9, *σ* = 1·4)	H1N1pdm	10	5·65	3·55–7·75		
	H3N2	1	1·60	−		
	H1N1	3	6·17	0–13·10	0·887	0·478
	B	1	3·00	−		
	Total	15	5·31	3·72–6·90		
Delta distribution (*Tc* = 2·9)	H1N1pdm	10	8·64	3·38–13·89		
	H3N2	1	1·63	−		
	H1N1	3	9·30	0–24·81	0·465	0·713
	B	1	3·33	−		
	Total	15	7·95	4·22–11·68		
Gamma distribution Gamma(4·2,0·68)	H1N1pdm	10	5·14	3·29–6·99		
	H3N2	1	1·58	−		
	H1N1	3	5·58	0–11·57	0·876	0·483
	B	1	2·84	−		
	Total	15	4·84	3·44–6·23		

*T*c, Mean generation interval; *σ*, standard deviation; *R*, reproduction number; CI, confidence interval; *F*, a statistic value in analysis of variance.

In the 15 outbreaks used for calculating *R*, we found that eight interventions, implemented individually or in combination, were employed after the reported outbreak date, except with social distance and antivirals like oseltamivir (see Table 5). These interventions included five non-pharmaceutical (symptom surveillance, case isolation, health education, environment disinfection and class/grade/school closure) and three pharmaceutical (symptomatic treatment, prophylaxis, vaccination) interventions. Symptom surveillance was implemented in 14 outbreaks. Case isolation, symptomatic treatment, health education and environment disinfection were employed in each outbreak. During four outbreaks (three H1N1pdm and one B subtype), class or grade closure was employed because cluster cases occurred in the class or grade, and school closure was employed in nine outbreaks (six H1N1pdm and three H1N1). Vaccination was employed in two outbreaks (one H1N1pdm and one H3N2). Prophylaxis was only employed in one H1N1 outbreak.

**Table 5.** The interventions in each outbreak and the reproduction number based on the epidemic model (n = 15)

Outbreak ID	*R*	*R*_con_	Symptom surveillance	Isolation	Symptomatic treatment	Prophylaxis	Health education	Environment disinfection	Vaccination	Social distance	Class/grade closure	School closure
1	2·87	0·00	Yes	Yes	Yes	No	Yes	Yes	No	No	No	No
8	2·28	0·87	Yes	Yes	Yes	No	Yes	Yes	No	No	Yes (7 days)	Yes (7 days)
9	2·58	0·99	Yes	Yes	Yes	No	Yes	Yes	No	No	Yes (8 days)	Yes (12 days)
10	2·33	0·00	No	Yes	Yes	No	Yes	Yes	No	No	No	Yes (7 days)
14	2·91	0·00	Yes	Yes	Yes	No	Yes	Yes	No	No	No	No
17	3·01	0·00	Yes	Yes	Yes	No	Yes	Yes	No	No	No	No
21	4·11	0·00	Yes	Yes	Yes	No	Yes	Yes	No	No	No	No
27	3·12	0·55	Yes	Yes	Yes	No	Yes	Yes	No	No	Yes (7 days)	Yes (10 days)
31	3·08	0·14	Yes	Yes	Yes	No	Yes	Yes	No	No	No	Yes (7 days)
38	1·48	0·00	Yes	Yes	Yes	No	Yes	Yes	Yes	No	No	Yes (4 days)
39	1·45	0·00	Yes	Yes	Yes	No	Yes	Yes	Yes	No	No	No
42	3·61	0·00	Yes	Yes	Yes	Yes	Yes	Yes	No	No	No	Yes (7 days)
43	2·12	0·26	Yes	Yes	Yes	No	Yes	Yes	No	No	Yes (5 days)	No
45	2·72	0·00	Yes	Yes	Yes	No	Yes	Yes	No	No	No	Yes (5 days)
46	2·51	0·00	Yes	Yes	Yes	No	Yes	Yes	No	No	No	Yes (4 days)

ID, Identification; *R*, reproduction number; *R*_con_, reproductive number with control measures.

According to whether the intervention was employed or not, we divided the 15 outbreaks into employed groups and non-employed groups. Then we compared the mean *R* of the two groups to infer if the intervention was employed based on a high *R*. There was no significance of class/grade closure (*t* = −0·492, *P* = 0·631), school closure (*t* = −0·286, *P* = 0·780) or prophylaxis (*t* = −1·405, *P* = 0·184). On the other hand, there was significance of vaccination (*t* = 3·479, *P* = 0·004), but before the countermeasure was chosen, the *R* of the employed group was lower than that of the non-employed group. These results reveal that the intervention was not employed based on whether the value of *R* was high or not.

**Fig. 1.**
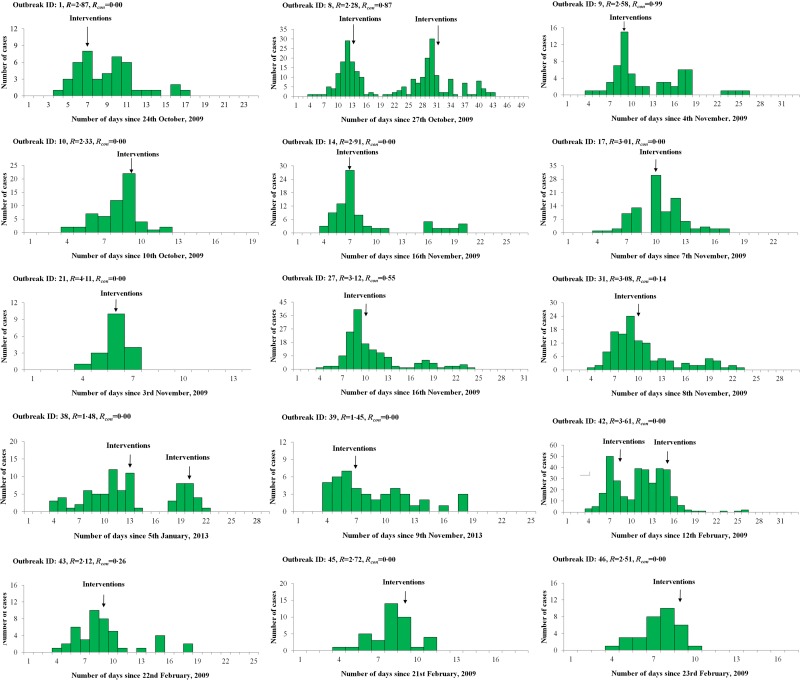
Epidemic curves of 15 influenza outbreaks used for calculating *R* based on an epidemic model in Changsha city, China. ID, Identification; *R*, reproduction number; *R*_con_, reproductive number with control measures.

The reproductive number with control measures (*R*_con_) of each outbreak was calculated by the epidemic model using the reported data which was collected after the reported date. We observed that *R*_con_ was <1 after the interventions were implemented and most *R*_con_ were 0 (see Table 5 and Fig. 1).

## DISCUSSION

Our results reveal that the mean value of *R* in small-scale outbreaks was higher than in large-scale ones (outbreaks which occurred in a neighbourhood, city or province) in which *R* was <2·0 [2–5, 9, 13, 14], whether or not *Tc* = 2·7 (*t* = 3·068, *P* = 0·007) or *Tc* = 2·9 (*t* = 3·494, *P* = 0·003), as calculated by the epidemic model. This implies that the attack rate and the peak incidence of a small-scale outbreak may be higher than those occurring in a city. The reasons for this difference are not clear, but may be caused by the high population density and contact frequency in a school or a prison compared to a neighbourhood. This may alert us to employ different countermeasures to control small-scale outbreaks rather than outbreaks occurring in a city at large.

Furthermore, the *R* values estimated by normal distribution, delta distribution, and gamma distribution models were higher than those calculated by the epidemic model. The former three models resulted in a higher s.d. and larger ranges of *R*, which might make the estimation more discrete and unstable. We conclude that the former three models seem to more easily overestimate the *R* of an influenza outbreak. And this overestimation may result in the control strategies being implemented excessively when the primary health department makes an emergency response policy. Thus, in the four models of the intrinsic growth rate method, the epidemic model might be the best one to recommend to estimate the transmissibility of influenza at the early stage of an outbreak.

We found that the commonly used interventions were symptom surveillance, case isolation, symptomatic treatment, health education and environment disinfection, followed by class/grade/school closure, vaccination and prophylaxis. They were not employed based on the transmissibility (the reproduction number *R*) of the outbreak. Although the transmissibility of most of the outbreaks decreased rapidly after the interventions, some of them were still close to 1, which would prolong an outbreak. Therefore, we recommend that local CDCs calculate the reproduction number of the outbreak by using the epidemic model and the data when they receive an outbreak report, thereby avoiding implementing countermeasures blindly.

It should be noted that this study has some limitations because of the lack of integrity of the data and the methodology of the model. Regarding the data we collected, epidemic curves of seven outbreaks were missing or not integrated, and there were 19 outbreaks showing no exponential growth in early stages, which meant that there was still more effort required from the primary health department to improve the quality of the data collection. If the data is collected more accurately through field investigation, the *R* estimation will be shown to be more precise. On the other hand, the epidemic model requires that the early stage of an outbreak must be ⩾3 days. The lower time span (especially <3 days) of the data may make the estimation unstable and unreliable. Finally, the epidemic model is only suitable to estimate the *R* of the outbreak in the early stage according to the principle of the model, which signified that we could not use the whole data if the outbreak was reported after the early stage. Therefore, after the exponential growth stage, particularly for the stage after the epidemic peak of the outbreak, other more complicated models should be utilized, such as an ordinary differential equation model or a stochastic individual-based model to conduct the transmissibility estimation.

In conclusion, we have shown the mean value of *R* of influenza outbreaks in Changsha, China, and our findings may show an appropriate example for the primary health department to assess the transmissibility of small-scale influenza outbreaks with a practical, yet accurate model at the early stage.

## References

[1] Chen T, Risk of imported Ebola virus disease in China. Travel Medicine and Infectious Disease 2014; 12: 650–658. 25467086 10.1016/j.tmaid.2014.10.015

[2] Liu R, The effectiveness of age-specific isolation policies on epidemics of influenza A (H1N1) in a large city in Central South China. PLoS ONE 2015; 10: e0132588. 26161740 10.1371/journal.pone.0132588 PMC4498797

[3] Longini IM Jr., Containing pandemic influenza at the source. Science 2005; 309: 1083–1087. 16079251 10.1126/science.1115717

[4] Longini IM Jr., Containing pandemic influenza with antiviral agents. American Journal of Epidemiology 2004; 159: 623–633. 15033640 10.1093/aje/kwh092

[5] Yang Y, The transmissibility and control of pandemic influenza A(H1N1) virus. Science 2009; 326: 729–733. 19745114 10.1126/science.1177373 PMC2880578

[6] Chen SC, Liao CM. Modelling control measures to reduce the impact of pandemic influenza among schoolchildren. Epidemiology and Infection 2008; 136: 1035–1045. 17850689 10.1017/S0950268807009284 PMC2870896

[7] Heffernan JM, Smith RJ, Wahl LM. Perspectives on the basic reproductive ratio. Journal of the Royal Society, Interface 2005; 2: 281–293. 10.1098/rsif.2005.0042 PMC1578275 16849186

[8] Wallinga J, Lipsitch M. How generation intervals shape the relationship between growth rates and reproductive numbers. Proceedings of the Royal Society of London, Series B: Biological Sciences 2007; 274: 599–604. 17476782 10.1098/rspb.2006.3754 PMC1766383

[9] Trichereau J, Estimation of the reproductive number for A(H1N1)pdm09 influenza among the French armed forces, September 2009–March 2010. Journal of Infection 2012; 64: 628–630. 22343067 10.1016/j.jinf.2012.02.005

[10] McBryde E, Early transmission characteristics of influenza A(H1N1)v in Australia: Victorian state, 16 May–3 June 2009. Eurosurveillance 2009; 14: pii: = 19363. 10.2807/ese.14.42.19363-en 19883544

[11] Hahné S, Epidemiology and control of influenza A(H1N1)v in the Netherlands: the first 115 cases. Eurosurveillance 2009; 1: pii: = 19267. 10.2807/ese.14.27.19267-en 19589332

[12] Chen T, Investigation of key interventions for shigellosis outbreak control in China. PLoS ONE 2014; 9: e95006. 24736407 10.1371/journal.pone.0095006 PMC3988114

[13] Germann TC, Mitigation strategies for pandemic influenza in the United States. Proceeding of the National Academy of Sciences USA 2006; 103: 5935–5940. 10.1073/pnas.0601266103 PMC1458676 16585506

[14] Tracht SM, Valle SYD, Hyman JM. Mathematical modeling of the effectiveness of facemasks in reducing the spread of novel influenza A(H1N1). PLoS ONE 2010; 5: e9018. 20161764 10.1371/journal.pone.0009018 PMC2818714

